# Digital Health Interventions by Clinical Pharmacists: A Systematic Review

**DOI:** 10.3390/ijerph19010532

**Published:** 2022-01-04

**Authors:** Taehwan Park, Jagannath Muzumdar, Hyemin Kim

**Affiliations:** 1 Pharmacy Administration and Public Health, College of Pharmacy and Health Sciences, St. John’s University, Queens, NY 11439, USA; muzumdaj@stjohns.edu; 2 College of Pharmacy and Health Sciences, St. John’s University, Queens, NY 11439, USA; hye.kim16@my.stjohns.edu

**Keywords:** clinical pharmacists, digital interventions, patient outcomes, randomized controlled trials

## Abstract

Integrating digital interventions in healthcare has gained increasing popularity among clinical pharmacists (CPs) due to advances in technology. The purpose of this study was to systematically review CP-led digital interventions to improve patients’ health-related clinical outcomes. PubMed and the Cochrane Database were searched to select studies that had conducted a randomized controlled trial to evaluate clinical outcomes in adults following a CP-led digital intervention for the period from January 2005 to August 2021. A total of 19 studies were included in our analysis. In these 19 studies, the most commonly used digital intervention by CPs was telephone use (*n* = 15), followed by a web-based tool (*n* = 2) and a mobile app (*n* = 2). These interventions were provided to serve a wide range of purposes in patients’ outcomes: change in lab values (e.g., blood pressure, HbA1c) (*n* = 23), reduction in health service use (*n* = 8), enhancing adherence (*n* = 6), improvement in drug-related outcomes (*n* = 6), increase in survival (*n* = 3), and reduction in health-related risk (e.g., CVD risk) (*n* = 2). Although the impacts of telephone-based interventions on patients’ outcomes were decidedly mixed, web-based interventions and mobile apps exerted generally positive influences. To date, little research has investigated the cost-effectiveness of digital interventions. Future studies are warranted.

## 1. Introduction

The last several decades have witnessed seismic advances in information technology. Technology evolution has facilitated the integration of digital technology into healthcare. For example, healthcare professionals (HCPs) now use their mobile devices for their work in daily practices [1]. Using their smartphone or tablet computer, HCPs can easily access patient charts, communicate with patients, and monitor disease management [2,3,4]. With the increasing use of smartphones, a number of HCPs have also adopted mobile applications (hereafter, ‘mobile apps’) in their practice. Approximately 77% of medical school healthcare professionals and students reported using at least one mobile app regularly, with 50% using their favorite app daily [5]. Mobile devices and apps are extensively used among HCPs during the conduct of their healthcare provision primarily because they are portable and easily accessible. In addition, digital healthcare provides benefits to patients when HCPs can provide timely interventions from a remote location. In this regard, digital healthcare is particularly helpful for individuals living in rural areas who may have limited access. Furthermore, technology-integrated interventions can benefit the elderly who may have limited personal and societal resources. Studies have shown that digital intervention resulted in health benefits [6] and an increase in digital competences [7] among older adults. For all of these reasons, integrating digital technologies in healthcare has attracted increasing attention.

As HCPs, pharmacists have shown interest in using digital technology in their practices [8,9,10]. For example, pharmacists have used personal digital assistance (PDA) as a tool for documenting their interventions, performing health assessments, providing patient education, and monitoring patient outcomes [11,12]. Pharmacists can also use systems with video cameras to approve prescriptions written at a remote site [13]. Each of these prior studies provided piecemeal evidence that showed how pharmacists employed digital technology in their practice.

Recently, Crilly and Kayyali systematically reviewed telehealth and digital technology used by community pharmacists [14]. Their study found that community pharmacists most often used the telephone as a digital intervention tool, followed by a remote monitoring device, a mobile app, and photo-aging software. These tools had been used for increasing vaccine rates, smoking cessation, hypertension management, and medication adherence. The study findings revealed that community pharmacists’ digital interventions had positive impacts on health outcomes in general. As their study focused on community pharmacists, no study has systematically examined the types of digital interventions delivered by clinical pharmacists. In addition, the impacts of such interventions by clinical pharmacists on patients’ clinical outcomes are yet to be systematically investigated. Therefore, the objective of this study was to perform a systematic review to evaluate how clinical pharmacists have used digital technologies to improve patient outcomes.

## 2. Materials and Methods

### 2.1. Search Strategy

To assess the current state of the literature on clinical pharmacist-led digital interventions, a systematic review of published studies was conducted. Specifically, we reviewed studies to evaluate how clinical pharmacists have used digital health to improve clinical outcomes for adult patients. Accordingly, we performed online searches of PubMed and the Cochrane Database of Systematic Reviews for the period from January 2005 to August 2021. We included only full-text articles published in English in peer-reviewed journals. To develop our search strategy, the patient, intervention, comparison, outcome (PICO) framework was used for identifying medical subject heading (MeSH) terms and keywords. Studies were included in our review if they contained:

P: Adult patients in randomized controlled trials (RCTs)

I: Digital intervention(s) by clinical pharmacists

C: Usual care (i.e., comparator treatment in the published studies)

O: Health-related clinical outcomes from patients

MeSH terms and keywords for clinical pharmacy included: pharmacist*, pharmacy, pharmacies, clinical pharmacy service*, and clinical pharmacy intervention*. MeSH terms and keywords for digital intervention(s) included: digital health, m-health, mobile health, telehealth, telemedicine, telecare, teleconsult*, telecommunicate*, telemonitor*, ehealth, electronic health, ecommunicate*, remote consultation, sms, short messaging service, text message*, internet consultation, internet monitoring, internet communicate*, video consultation, video monitoring, video communicate*, *phone, sensor*, and wearable.

### 2.2. Selection Process and Inclusion/Exclusion Criteria

**Figure 1:**
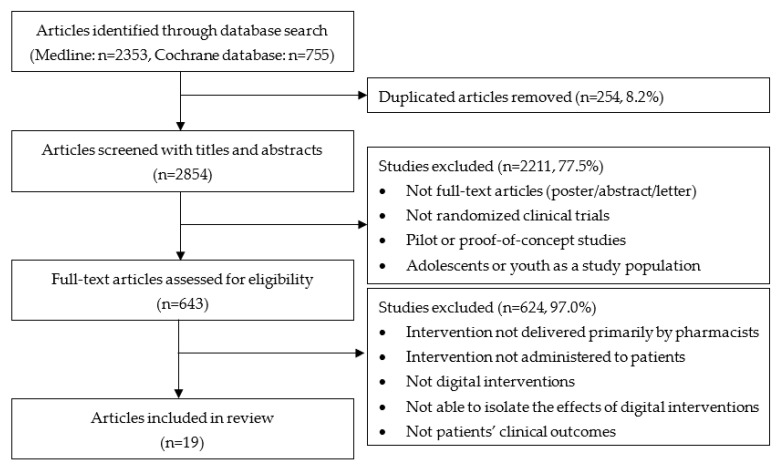
Flowchart of the search strategy and selection of articles.

Figure 1 depicts a flowchart showing the process of selecting the relevant articles. Our database search identified 2353 and 755 articles from PubMed and the Cochrane Database, respectively. After removing duplicates, there was a total of 2854 unique articles. Titles and abstracts of these articles were reviewed for the first level of screening. Studies were excluded if (a) they were not full-text articles (e.g., poster or letter); (b) they did not include RCTs; (c) they were pilot or proof-of-concept studies; and (d) the study populations did not include adults (i.e., adolescents or youth). This screening process resulted in the exclusion of 2211 articles. In the second level of screening, the remaining full-text articles were assessed for eligibility. Studies were excluded if (a) interventions were not delivered primarily by pharmacists; (b) interventions were not administered to patients; (c) digital interventions were not included; (d) the effects of the digital intervention were not able to be isolated; and (e) clinical outcomes from patients were not measured. Following discussions, we determined to include articles if the study outcomes were healthcare service use (e.g., use of drug(s), ED visit, hospitalization), adherence to medication or a clinical guideline, drug-related outcomes (e.g., incidence and severity of adverse events), and health-related risk (e.g., CVD risk, smoking) because these outcomes could be strongly related to patients’ clinical outcomes. After application of these exclusion criteria, only 19 articles remained which were included in our analysis.

Data from the 19 articles were extracted by two reviewers (TP and HK). Using these data, the reviewers created a template including the following variables: author(s) and year of publication; study design (setting, subjects, intervention, control); outcome(s); and results.

To evaluate the methodological quality of the RCTs included in our review, we used the Jaded scale [15] and the PEDro scale [16]. A score on the Jaded scale (ranging from 0 to 5) is determined based on the level of randomization, blinding, and withdrawal/drop-out in each RCT. A score of 0 to 1 indicates a high risk of methodological bias; a score of 2 to 3 represents a moderate risk; and a score of 4 to 5 suggests a low risk. As the PEDro scale was developed to evaluate physiotherapy clinical trials, we adjusted one original item (“there was blinding of all therapists who administered the therapy”) to the study context (“there was blinding of all pharmacists who administered the intervention”) when assessing the quality of the studies included in our review. We considered a score of 0 to 3 on the PEDro scale to be a high risk of bias; a score of 4 to 6 to be a moderate risk; and a score of 7 to 10 to be a low risk.

## 3. Results

Table 1 shows the summary of the included 19 studies. The majority of these studies were conducted in the U.S. (*n* = 15). The types of digital interventions were telephone-based care (*n* = 15), web-based monitoring (*n* = 2), and mobile app use (*n* = 2). These interventions were provided to serve a wide range of purposes in patients’ outcomes: change in lab values (e.g., blood pressure, HbA1c) (*n* = 23), reduction in health service use (*n* = 8), enhancing adherence to medication or a clinical guideline (*n* = 6), improvement in drug-related outcomes (*n* = 6), increase in survival (*n* = 3), and reduction in health-related risk (e.g., CVD risk, smoking) (*n* = 2).

### 3.1. Telephone-Based Intervention

A total of 15 studies used a telephone as an intervention tool by clinical pharmacists. The impacts of the phone-based interventions on patients’ outcomes were mixed. Of these 15 studies, five studies reported significant effectiveness of the phone interventions [17,18,19,20,21]. Specifically, such interventions showed positive effects on lowering serum uric acid (sUA) levels among individuals with gout [17], the number of drug-related problems among those visiting outpatient cardiology clinics [18], mortality among those using five or more prescription drugs [19], and improved control of both BP [20] and international normalized ratio (INR) among patients discharged on warfarin [21]. However, five other studies showed no significant differences in outcomes between the treatment group who received phone-based interventions and the control group receiving usual care [22,23,24,25,26]. Specifically, no significant differences were reported between these two groups in these studies with respect to the proportion of individuals who attempted tobacco cessation among tobacco users [22], adherence to cancer drug for almost all cycles, overall survival (OS), and progression-free survival (PFS) among individuals with metastatic colorectal or gastric cancer [23], changes in HbA1c level and adherence to diabetic drugs [24], adherence to cardiovascular drugs and the percent of those with LDL-C goal achievement among individuals with coronary heart disease (CHD) [25], and the proportion of emergency department (ED) visits and readmission to hospital within 30 days of discharge among those with cancer [26]. Five studies revealed inconsistent effects of phone intervention on patients’ outcomes [27,28,29,30,31]. In other words, the interventions in each of these studies resulted in improvement in some outcomes, but not in others. For example, Bosworth et al. found that telephone intervention by clinical pharmacist specialists significantly reduced total cholesterol at 6 months in the treatment group, but no significant differences were found in CVD risk, sBP, dBP, LDL, HDL, BMI, HbA1c at 6 or 12 months, and total cholesterol at 12 months between the treatment and the control groups [27]. In Carter et al.’s study, telephone-based medication therapy management (MTM) services significantly improved individuals’ adherence to the American Heart Association (AHA)’s clinical guidelines, but their levels of BP, HbA1c, and lipids were not significantly different compared to those receiving usual care [28]. Similarly, Choudhry reported that phone-based consultation resulted in improvement in medication adherence, but the impacts of this phone intervention on disease control based on LDL, sBP, HbA1c, hospitalization, and physician office visits were not significant [29]. Gernant et al. revealed no significant differences in 60-day ED utilization between the treatment group receiving phone-based MTM services and the control group receiving usual nursing care, but reported significantly lower ED use among individuals in the lowest risk quartile in the treatment group compared to the control group [30]. Zillich et al. also demonstrated no significant differences in 30-day and 60-day hospitalizations between the phone-based MTM group and the usual home care group, but found significantly lower 30-day and 60-day hospitalizations among those in the lowest risk quartile in the treatment group [31].

### 3.2. Web-Based Intervention

Two studies examined the impacts of web-based interventions by clinical pharmacists on patients’ outcomes [32,33]. In both studies, the interventions exerted positive influences on most of the outcomes examined. In Green et al.’s study, the treatment group received home BP monitoring and web services as well as web-based communications with pharmacists while the control group received only either home BP monitoring plus web services or usual care [32]. This study found significant improvements in most of the outcomes such as sBP, dBP, the percent of individuals with controlled BP (<140/90 mmHg), and the number of antihypertensive agents and aspirin used in the treatment group. They found no significant differences only in BMI change between the two groups. In Magid et al.’s study, the treatment group received web-based BP monitoring and education by pharmacists whereas the control group received usual care [33]. They also reported significant improvements in most of the outcomes—i.e., achievement of BP goal, sBP, dBP, and antihypertensive medication intensity; however, adherence to antihypertensive medications was not significantly different between the two groups.

### 3.3. Mobile-Based Intervention

Two studies from the same clinical trial included a mobile-based intervention to investigate the impact of this intervention on patients’ outcomes [34,35]. In this trial, a mobile app was developed to monitor and manage medication therapy for kidney transplant recipients. In these two studies, the intervention group received the mobile app-based intervention whereas the control group received usual care. The studies showed significant reductions in medication errors, incidences of grade 3 or higher adverse events (AEs), hospitalization [34], and tacrolimus intrapatient variability (IPV) in the treatment group [35]. However, no significant differences were found in incidences of grade 1 or 2 AEs and the infection rates between the two groups [35].

Overall, all the included studies were considered to have a low risk of methodological bias (*n* = 11 and 14) or a moderate methodological risk of bias (*n* = 8 and 5) using the Jaded scale and PEDro scale, respectively. That is, no study was considered to have a high risk of methodological bias.

## 4. Discussion

We systematically reviewed studies that evaluated the impacts of clinical pharmacist-led digital interventions on patients’ clinical outcomes. In these studies, clinical pharmacists used telephones, web tools, and mobile apps for their digital interventions. Overall, the impacts of telephone-based interventions in the studies were inconsistent: five studies showed benefits from these interventions, another five studies revealed no significant effects, and the remaining five studies reported mixed effects from the interventions. Web-based interventions resulted in positive impacts on changes in lab values (e.g., sBP, dBP) and health service use (e.g., antihypertensive drug and aspirin use) in patients with hypertension. Clinical pharmacists’ use of mobile apps significantly improved drug-related outcomes (e.g., reduction in medication errors and severe AEs) and health service use (e.g., reduction in hospitalizations) in kidney transplant recipients. When the study results were analyzed by the types of outcomes, we found that digital interventions were generally effective in lowering health service use (e.g., hospitalization, drug use) and improving drug-related outcomes (e.g., medication errors, AEs). However, they did not always result in significant improvements in other outcomes such as changes in lab values, adherence, survival, and health-related risk.

Our study found that telephoning was the most frequently used intervention tool among clinical pharmacists. This result is consistent with earlier findings obtained from community pharmacists [14]. Our review found that in 15 out of 19 studies (78.9%), clinical pharmacists used a telephone as a digital intervention tool. Similarly, a previous study reported that nine out of 13 studies (69.2%) used a telephone as the digital intervention tool by community pharmacists. As such, both clinical pharmacists and community pharmacists used telephones as the most commonly used intervention tool. Telephone use represents a somewhat traditional approach of digital interventions. In only a limited number of studies, other types of digital technology such as mobile apps were used by clinical pharmacists [34,35] and community pharmacists [36]. No studies used social media, a wearable device, or video conferencing as a digital intervention by pharmacists. As noted previously, approximately 77% of medical school healthcare professionals and students reported using at least one mobile app regularly, with 50% using their favorite app daily [5]. Physicians use medical apps for many reasons such as searching relevant literature, accessing patient charts, submitting electronic prescriptions, and monitoring disease management [2,3,4]. Pharmacists can also consider more novel technologies such as mobile apps, social media, and wearable devices for their patients where pharmaceutical care is provided. By using more diverse technologies, pharmacists could improve patients’ outcomes in additional domains.

The current study’s findings highlight a wide range of applications of clinical pharmacists’ interventions to diverse study populations. The pharmacists provided their interventions to extensive populations such as patients with CHD, hypertension, hyperlipidemia, diabetes, cancer, gout, kidney conditions, tobacco use, using warfarin, and those using five or more drugs for chronic conditions. Accordingly, the purposes of providing these interventions were also very comprehensive, ranging from improving lab values (e.g., blood pressure, HbA1c), adherence, drug-related outcomes, and survival to lowering health service use and health-related risks. Of note, community pharmacists used digital interventions to serve somewhat limited purposes. Crilly and Kayyali found that community pharmacists’ interventions focused primarily on improvement in medication counseling and adherence, which represents traditional roles of the community pharmacist [14]. Crilly et al. argued that community pharmacists using more diverse technologies such as social media and mobile apps could help define their roles for more diverse outcomes/domains such as weight management, sexual health, and alcohol use [37]. Findings from both our study and their study suggest that digital interventions by clinical pharmacists are more likely to serve more various purposes compared to those interventions by community pharmacists. Nevertheless, community pharmacists are easily accessible professionals who routinely encounter people in their community. In this sense, digital care services are conveniently designed and efficiently delivered by community pharmacists, which can ultimately result in positive outcomes for patients.

Although digital interventions have gained increasing popularity, there has been little investigation into the cost-effectiveness (CE) of these interventions. Accordingly, whether a digital intervention is cost-effective remains to be elucidated. For example, Pyne et al. investigated the cost-effectiveness of a telemedicine-based collaborative care intervention for individuals with depression in rural areas [38]. They found the incremental cost-effectiveness ratio (ICER) of this intervention to be about $86,000/quality-adjusted life year (QALY) gained, which suggests that the intervention may or may not be cost-effective depending on the cost-effectiveness threshold. Another study conducted by Painter et al. evaluated the cost-effectiveness of telemedicine-based collaborative care for veterans with posttraumatic stress disorder (PTSD) in rural areas [39]. The ICER for the telemedicine intervention was about $186,000/QALY gained. This ICER value was higher than the conventionally reported cost-effectiveness thresholds of $50,000–$150,000/QALY gained, deeming the intervention cost-ineffective. However, their analyses focusing on patient subgroups with comorbidities such as depression, anxiety, and panic disorder concluded that this intervention was cost-saving for these groups. Regarding the CE study of a pharmacist-led intervention, Avery et al. performed a cost-effectiveness analysis of a pharmacist’s information technology intervention composed of feedback, educational outreach, and dedicated support for individuals with medication errors [40]. Their analysis resulted in an ICER of £66/medication error avoided. As they did not quantify the economic effects on patients’ quality of life, the ICER with the QALY gained could not be generated. Thus, they were not able to assess whether the ICER with medication errors avoided for this intervention would be considered cost-effective according to the policy decision rules in England that require the ICER with the QALY gained.

To our knowledge, this is the first systematic review of the impacts of digital interventions by clinical pharmacists on patients’ outcomes. Our review demonstrated that a clinical pharmacist-led digital intervention has the potential to benefit patients. There are several limitations to this study. First, because we included only articles published in English, there may be selection bias. If prior studies exist published in non-English languages, a future review could consider including these studies. Second, we were not able to pool different outcomes quantitatively because of heterogeneity inherent to these outcomes. Therefore, we narrated the findings of the studies qualitatively. In addition, most of the included studies were conducted in the U.S., which could limit the generalizability of the study findings. Finally, although we endeavored not to miss any relevant articles by following the recommendations of the Preferred Reporting Items for Systematic Reviews and Meta-Analyses (PRISMA) statement, there may have been articles not captured by our search with the two databases employed in this study.

In summary, clinical pharmacists’ digital interventions were limited to the use of telephones, web tools, and mobile apps. In future studies, clinical pharmacists should consider novel technologies such as social media and wearable devices for patients who receive pharmaceutical care. Additionally, future studies need to be carefully designed by taking the contents of the intervention and the study population into account because these aspects are strongly related to the outcomes of the intervention.

## 5. Conclusions

Previous studies have shown that the telephone has been the most commonly used intervention tool among clinical pharmacists, followed by web-based interventions and mobile apps. Our review found that the impacts of telephone-based interventions on patients’ outcomes were not consistent. Therefore, pharmacists should be prudent in developing a telephone-based intervention by considering, for example, the study population, the structure of the intervention, and the contents delivered by the intervention. Impacts of web-based interventions and mobile apps were generally positive, which suggests the benefits of continued use of these tools. However, more studies are warranted because of the limited studies using these tools as interventions. Additionally, only limited evidence exists regarding the cost-effectiveness of digital interventions. Therefore, future research is needed to first identify the economic value of the interventions and then implement the cost-effective interventions.

## Figures and Tables

**Table 1:** Characteristics of the articles evaluating digital interventions by clinical pharmacists.

Author (Year)	Setting (Duration and Location)	Subject	Intervention (*n*, Mean Age, %Female)	Control (*n*, Mean Age, %Female)	Outcomes	Result	Risk of Bias Using the Jaded/PEDro Scales
Telephone-based intervention							
Adams et al. (2015) [22]	Three months, privately insured population in the U.S.	Tobacco users who were enrolled in Clinical Pharmacy Cardiac Risk Service (CPCRS)	Telephone-based counseling (*n* = 64, 66.6, 43.8%)	Usual care (*n* = 56, 64.6, 28.6%)	Primary: Proportion of individuals who reported a tobacco cessation attempt during follow-up Secondary: Proportion of individuals who had (1) at least one contact with a phone counseling service; (2) purchased at least one tobacco cessation medication aid; and (3) attended at least one tobacco cessation program or webinar	Primary: No significant difference in tobacco cessation attempt between the treatment and the control groups (38.6% vs. 36.2%, *p* = 0.804) Secondary: No significant difference between the two groups in the proportion of individuals who had (1) at least one contact with a phone counseling service (0.0% vs. 5.4%, *p* = 0.099); (2) purchased at least one tobacco cessation medication aid (10.9% vs. 19.6%, *p* = 0.183); and (3) attended at least one tobacco cessation program or webinar (1.6% vs. 0.0%, *p* = 0.348)	Low/Low
Bosworth et al. (2018) [27]	12 months, a veterans’ medical center in the U.S.	Patients with hypertension and/or hypercholesterolemia	Telehealth intervention by clinical pharmacist specialists (*n* = 215, 60.9, 15.3%)	Educational control (*n* = 213, 61.5, 15.0%)	Primary: Framingham cardiovascular disease (CVD) risk score at 6 and 12 months Secondary: systolic blood pressure (sBP), diastolic blood pressure (dBP), total cholesterol, low-density lipoprotein (LDL), high-density lipoprotein (HDL), body mass index (BMI), HbA1c (for those w/diabetes) at 6 and 12 months	Primary: No significant differences in Framingham CVD risk score, sBP, dBP, LDL, HDL, BMI, HbA1c at 6 or 12 months and total cholesterol at 12 months Secondary: Significant decline in total cholesterol at 6 months in the treatment group compared to the control group (difference: −7.0, 95%CI: −13.4 to −0.6, *p* = 0.03)	Moderate/Moderate
Carter et al. (2018) [28]	12 months, physician offices and health centers in the U.S.	Patients with diabetes or hypertension	Telephone-based medication therapy management (MTM) (*n* = 149, 63.7, 46.3%)	Usual care (*n* = 153, 64.1, 52.9%)	Primary: Adherence to the American Heart Association (AHA)’s guideline developed for individuals with CV conditions Secondary: Changes in key CV risk factors such as blood pressure, HbA1c, and lipids	Primary: Significant improvement in adherence to the guideline only in the treatment group (*p* = 0.02) Secondary: No significant differences in the secondary outcomes between the treatment group and the control group (*p*-values ranging from 0.06 to 0.73)	Moderate/Moderate
Choudhry (2018) ^a^ [29]	12 months, primary care practice sites in the U.S.	Patients with hyperlipidemia, hypertension, and diabetes	Telephone-based consultation (*n* = 2038, 60.4, 45.3%)	Usual care (*n* = 2040, 59.2, 45.0%)	Primary: Medication adherence measured by proportion of days covered (PDC) Secondary: (1) Disease control based on achieved levels of LDL, sBP, and HBA1c for at least one condition and all conditions as well as (2) healthcare service use	Primary: Significantly higher improvement in medication adherence in the treatment group compared to the control group (difference = 4.7%, 95% CI: 3.0–6.4%) Secondary: No significant difference between the two groups in (1) achieving disease control for at least one condition (OR = 1.10, 95% CI:0.94–1.28) and all conditions (OR = 1.05, 95% CI: 0.91–1.22) as well as (2) hospitalization (OR = 1.02, 95% CI: 0.78–1.34) and physician office visits (OR = 1.11, 95% CI: 0.91–1.36) Significantly fewer emergency department visits in the treatment group compared to the control group (OR = 0.62, 95% CI: 0.45–0.85)	Low/Low
Eldeib et al. (2018) ^b^ [23]	12 months, National Cancer Institute in Egypt	Patients with metastatic colorectal or gastric cancer	Follow-up telephone call during the treatment cycles (i.e., from cycle 1 to cycle 12) (*n*= 44, 50.0, 63.6%)	Standard care (*n* = 38, 44.8, 63.2%)	Primary: Medication adherence measured by the pill count method Secondary: Overall survival (OS) and progression-free survival (PFS)	Primary: No significant difference in medication adherence between the treatment group and the control group for all cycles (98.99% vs. 96.83%, *p* = 0.354) except for the 11th cycle (100% vs. 92.86%, *p* = 0.046) Secondary: No significant difference between the two groups in the median OS (10.13 in the treatment group vs. 8.10 in the control group, *p* = 0.84) and the median PFS (5.20 in the treatment group vs. 6.13 in the control group, *p* = 0.48)	Moderate/Moderate
Gernant et al. (2016) [30]	Two months, home health population in the U.S.	Medicare-insured patients admitted to the home health agencies (HHAs)	Telephone-based MTM (*n* = 297, 71, 58%)	Usual nursing care (*n* = 359, 73, 61%)	60-day all-cause emergency department (ED) utilization	No significant difference in 60-day ED utilization (24.4% in the treatment group vs. 25.1% in the control group, 95% CI: 0.79–1.57) However, significantly lower ED utilization among patients in the lowest risk quartile for the treatment group (OR = 2.52, 95% CI: 1.15–5.49, *p* = 0.02)	Low/Low
Goldfien et al. (2016) [17]	Six months, Kaiser Permanent Northern California patient population in USA	Patients with gout	Telephone-based program (*n* = 37, 60.9, 2.7%)	Usual care (*n* = 40, 58.0, 20.0%)	Primary: Achievement of a serum uric acid (sUA) level of 6.0 mg/dL or below Secondary: Mean change in sUA levels	Primary: Higher percent of achievement of sUA level at or below 6.0 mg/dL in the treatment group compared to the control group (35% vs. 13%, *p* = 0.03) Secondary: Significant change in mean sUA levels in the treatment group compared to the control group (−1.5 mg/dL vs. 0.1 mg/dL, *p* < 0.001)	Moderate/Moderate
Huiskes et al. (2019) [18]	One month, hospitals in the Netherlands	Patients visiting outpatient cardiology clinics	Telephone call (*n* = 90, 65.8, 44.4%)	Usual care (*n* = 85, 66.2, 37.6%)	Number of drug-related problems (DRPs) one month after visiting the cardiologist	Significant reduction in the number of DRPs in the treatment group compared to the control group (0.3 vs. 0.8, *p* < 0.001)	Moderate/Moderate
Lauffenburger et al. (2019) ^c^ [24]	12 months, privately insured population in the U.S.	Patients with diabetes	Telephone-based consultation (*n* = 700, 54.9, 34.6%)	Usual care (*n* = 700, 54.6, 39.8%)	Primary: Change in HbA1c from baseline Secondary: (1) Proportion of patients achieving HbA1c < 8%, and (2) medication adherence measured by PDC	Primary: No significant difference in HbA1c change between the two groups (difference = 0.06, 95% CI: −0.20 to 0.32) Secondary: (1) No significant difference in the proportion of those achieving HbA1c < 8% between the two groups (OR = 0.91, 95% CI: 0.71–1.17) (2) No significant difference in medication adherence between the two groups (OR = 0.92, 95% CI: 0.72–1.17)	Low/Low
Ma et al. (2010) [25]	12 months, medical center in the U.S.	Patients with coronary heart disease (CHD)	Telephone-based counseling (*n* = 351, 60.4, 40.2%)	Usual care (*n* = 338, 60.3, 40.2%)	Primary: Percent of patients with a serum LDL-C <100 mg/dL Secondary: Proportion of adherence to statin medication	Primary: No significant difference in the percent of individuals with LDL-C <100 mg/dL between the treatment and control groups (65% vs. 60%, *p* = 0.29) Secondary: No significant difference in adherence to statin in the two groups (0.88 vs. 0.90, *p* = 0.51)	Moderate/Low
Margolis et al. (2013) ^d^ [20]	Six to eighteen months, primary care clinics in the U.S.	Patients with hypertension	Telemonitoring (*n* = 228, 62.0, 45.2%)	Usual care (*n* = 222, 60.2, 44.1%)	Primary: Control of BP (sBP < 140 mmHg and dBP < 90 mmHg) at 6 and 12 months Secondary: BP control and change in BP at 18 months	Primary: Significant improvement in BP control in the treatment group compared to the control group at 6 or 12 months (all *p*-values < 0.001) Secondary: Significant improvement in the treatment group in BP control (*p* = 0.003) and sBP (*p* = 0.004), but marginally insignificant in dBP (*p* = 0.07) at 18 months	Low/Low
Salmany et al. (2017) [26]	One month, cancer center in the U.S.	Patients with cancer who were discharged from inpatient services	Follow-up telephone call after hospital discharge (*n* = 166, 47.2, 54.3%)	No follow-up call (*n* = 166, 49.2, 52.4%)	ED visits and readmission to hospital within 30 days of discharge	No significant differences between the treatment group and the control groups in ED visit (44% vs. 52%, *p* = 0.123) and hospital readmission (37% vs. 43%, *p* = 0.317) within 30 days of discharge	Low/Low
Sudas Na Ayutthaya et al. (2018) [21]	Three months, hospital in Thailand	Patients prescribed warfarin upon discharge	Telephone call (*n* = 25, 56.6, 72%)	Standard pharmacy services (*n* = 25, 58.7, 48%)	(1) Proportion of international normalized ratio (INR) values in range, (2) proportion of INR out of range, (3) percent of patients with one or more out-of-range INR values, and (4) time in therapeutic range (TTR)	(1) Significantly higher proportion of INR values in range in the treatment group compared to the control group (45.6% vs. 24.1%, *p* = 0.005) (2) Significantly lower proportion of INR out of range in the treatment group compared to the control group (11.4% vs. 24.1%, *p* = 0.037) (3) Significantly lower percent of those with one or more out-of-range INR values in the treatment group compared to the control group (84% vs. 100%, *p* = 0.037) (4) Significantly higher mean TTR in the treatment group compared to the control group (49.8 vs. 28.0, *p* = 0.017)	Moderate/Low
Wu et al. (2006) [19]	Three months, hospital in Hong Kong	Clinically stable patients with prescription of five or more drugs on at least two consecutive visits to clinic	Telephone call midpoint between the two clinic visits (*n* = 219, 71.2, 51.0%)	No telephone call(*n* = 223, 70.5, 52.0%)	All-cause mortality	Significant reduction in mortality in the treatment group compared to the control group (relative risk = 0.59, 95% CI: 0.35–0.97, *p* = 0.039)	Low/Low
Zillich et al. (2014) [31]	Two months, home healthcare centers in the U.S.	Medicare-insured home healthcare patients	Telephone-based MTM (*n* = 415, 73, 58%)	Usual home healthcare (*n* = 480, 73, 62%)	Primary: 60-day all-cause hospitalization Secondary: 30-day all-cause hospitalization	Primary: No significant difference in 60-day all-cause hospitalization between the two groups (*p* = 0.19) However, significant lower 60-day hospitalization in the lowest baseline risk quartile for the treatment group (*p* = 0.01) Secondary: No significant difference in 30-day all-cause hospitalization between the two groups (*p* = 0.30) However, significant lower 30-day hospitalization in the lowest risk quartile for the treatment group (*p* = 0.01)	Low/Low
Web-based intervention							
Green et al. (2008) [32]	12 months, medical centers in the U.S.	Patients with hypertension alone (no diagnosis of diabetes, CV or renal disease, or other serious conditions)	Web-based communications with a pharmacist and home BP monitoring and access to patient web services (*n* = 261, 59.3, 55.9%)	CTRL 1: Home BP monitoring and access to patient web services (*n* = 259, 59.5, 45.9%)CTRL 2: Usual care(*n* = 258, 58.6, 54.7%)	Primary: Changes in sBP, dBP, and the percent of patients with controlled BP (<140/90 mmHg) Secondary: Changes in the number of antihypertensive medications, aspirin use, and BMI	Primary: Significant improvement in changes in sBP, dBP, and the percent with controlled BP in the treatment group compared to the control groups (all *p*-values < 0.001) Secondary: Significantly more reductions in the number of antihypertensive agents and aspirin use in the treatment group compared to the control groups (all *p*-values < 0.05) However, no significant difference in BMI change between the treatment group and the control groups (difference: −0.9, 95% CI: −2.1 to 0.3)	Low/Low
Magid et al. (2013) [33]	Six months, privately insured population in the U.S.	Patients with hypertension	Web-based blood pressure monitoring and education (*n* = 175, 60.0, 38.3%)	Usual care (*n* = 173, 59.1, 41.0%)	Primary: Proportion of patients who attained their goal BP Secondary: Changes in sBP, dBP, antihypertensive medication intensity, and antihypertensive medication adherence measured by medication possession ratio (MPR)	Primary: Significantly higher proportion of patients achieving BP goal in the treatment group compared to the control group (RR = 1.5, 95% CI: 1.2–1.9) Secondary: Significantly higher changes in sBP, dBP, and antihypertensive medication intensity in the treatment group (all *p*-values < 0.01) No significant difference in MPR between the two groups (0.86 in the treatment group vs. 0.87 in the control group, *p* = 0.98)	Low/Low
Mobile-based intervention							
Fleming et al. (2021) [35]	12 months, university medical center in the U.S.	Kidney transplant recipients	Mobile application for monitoring and managing medication therapy (*n* = 68, 50.2, 48.5%)	Usual care (*n* = 68, 51.2, 38.2%)	Intrapatient variability (IPV)	Significant decrease in tacrolimus IPV in the treatment group compared to control group (*p* = 0.01)	Moderate/Low
Gonzales et al. (2021) [34]	12 months, university medical center in the U.S.	Kidney transplant recipients	Mobile application for monitoring and managing medication therapy (*n* = 68, 50, 49%)	Usual care (*n* = 68, 51, 38%)	Primary: Incidence and severity of medication errors and adverse events (AEs) Secondary: Infections and hospitalizations	Primary: Significant reduction in medication errors in the treatment group compared to the control group (incidence risk ratio (IRR) = 0.39, 95% CI: 0.28–0.55) No significant difference in incidence of grade 1 or grade 2 AEs between the two groups, but a significantly lower incidence of grade 3 or higher AEs in the treatment group (IRR = 0.55, 95% CI: 0.30–0.99) Secondary: No significant difference in infection rates between the two groups, but a significantly lower rate of hospitalizations in the treatment group (IRR = 0.46, 95% CI: 0.27–0.77)	Low/Low

^a,c^ Results based on the intention-to-treat analyses. ^b,d^ Results included only clinical outcomes.

## Data Availability

Not applicable.
